# Human herpesvirus 6B infection in an adult with hemophagocytic lymphohistiocytosis carrying an UNC13D mutation: a case report and literature review

**DOI:** 10.3389/fimmu.2025.1679372

**Published:** 2025-11-28

**Authors:** Pingping Xiao, Qingqing Wang, Tingting Li, Zhigao Dong, Junnan Su, Yongquan Chen

**Affiliations:** 1 Department of Hematology and Rheumatology, The Second Affiliated Hospital of Xiamen Medical College, Xiamen, China; 2 Department of Radiology, The Second Affiliated Hospital of Xiamen Medical College, Xiamen, China

**Keywords:** hemophagocytic lymphohistiocytosis, familial HLH, human herpesvirus 6B, UNC13D, case report

## Abstract

Hemophagocytic lymphohistiocytosis (HLH) is a life-threatening hyperinflammatory syndrome. The cause of onset broadly distinguishes primary from acquired HLH. However, an increasing number of carrying HLH gene mutation cases have been reported in adults, and the relationship between genetic alterations and the onset of HLH in adults is still being explored. In this case, a 43-year-old woman with a one-month history of abnormal liver function and fever presented for evaluation. Laboratory data indicated pancytopenia, elevated ferritin levels, decreased fibrinogen levels, and the presence of phagocytes in the bone marrow. She was diagnosed with HLH and found to have a heterozygous mutation in the *UNC13D* gene. The onset of symptoms in this patient coincided with the exacerbation of human herpesvirus 6B infection. From our review of case reports published in the past seven years, patients with HLH carrying this heterozygous gene mutation were diagnosed in adults. The patient remains alive and healthy after comprehensive treatment. The genetic background must not be overlooked in the etiological diagnosis of adult hemophagocytic lymphohistiocytosis, even in the presence of infectious pathogenic factors. Future studies need to be undertaken involving a larger number of cases, along with virology and genomics correlation.

## 1 Introduction

Hemophagocytic lymphohistiocytosis (HLH) has broad clinical manifestations, including fever, hepatitis, hepatosplenomegaly, cytopenia, high ferritin levels, coagulopathy, and hypertriglyceridemia. HLH occurs as a result of a “state of excessive immune activation” or uncontrolled immune responses. Mortality estimates range from 20%–88% (1). Familial HLH (FHL) is caused by biallelic defects in *PRF1*, *UNC13D*, *STX11*, or *STXBP2* that severely impair granule-mediated cytotoxicity, the process by which perforin/granzyme-containing granules fuse with the plasma membranes of natural killer (NK) cells or cytotoxic T lymphocytes to induce apoptosis in target cells (2). Acquired HLH, which occurs secondary to infection, malignancy, and rheumatic disease, has a poor prognosis and is fatal in 50%–75% of patients, with early death attributable to progressive multiorgan damage. Human herpesvirus 6B (HHV-6B) is a ubiquitous herpes virus that infects >95% of adults (3).Adult-onset infectious HLH associated with genetic mutations is relatively uncommon. Here, we report the case of a 43-year-old female patient with HLH and HHV-6B infection, in whom a heterozygous mutation in the *UNC13D* gene was identified. We also provide a review of recent reports on patients with HLH carrying heterozygous *UNC13D* mutations. Our case report aims to emphasize that the genetic background should not be overlooked in the etiological diagnosis of adult HLH, even when infectious pathogens are present. We also explored the potential association between UNC13D gene mutations, HHV-6B infection, and HLH in adults.

## 2 Case presentation

A 43-year-old woman presenting with a one-month history of abnormal liver function and fever was referred to our hospital. Upon physical examination, her vital signs were as follows: temperature, 39.2 °C; blood pressure, 136/94 mmHg; heart rate, 120 beats/min; and respiratory rate, 20 breaths/min. She did not present with sternal tenderness, superficial lymphadenopathy, hepatomegaly, or splenomegaly, and she had no relevant personal or family medical history.

Her laboratory blood test results at admission were as follows: hemoglobin level, 149.0 g/L (normal range [NR], 115–150 g/L); white blood cell count, 2.41 × 10^9^/L (NR, 4–10 × 10^9^/L); platelet count, 272 × 10^9^/L (NR, 125–350 × 10^9^/L); reticulocyte percentage, 1.95% (NR, 0.3–3.0%); serum glutamic oxaloacetic transaminase, 366.50 IU/L (NR, 13.0–35.0 IU/L); serum glutamic pyruvic transaminase, 265.60 IU/L (NR, 7.0–40.0 IU/L); serum γ-glutamyl transpeptidase, 175 IU/L (NR, 7.0–45.0 IU/L); and serum lactate dehydrogenase, 739.00 IU/L (NR, 100.0–240.0 IU/L). Serum alkaline phosphatase levels were elevated (177.80 IU/L; NR, 35–100 IU/L), and triglyceride levels were measured at 1.48 mmol/L (NR, 0.4–1.8 mmol/L). Coagulation function and erythrocyte sedimentation rate were also within the NR. She tested negative for Epstein–Barr virus (EBV), respiratory virus, and hepatitis virus. Results were negative for the antinuclear antibody, antiphospholipid antibody, anti-extractable nuclear antigen antibody, antineutrophil cytoplasmic antibody, rheumatoid factor, and anti-cyclic citrullinated peptide tests. Tumor indices, including carcinoembryonic antigen, alpha-fetoprotein, carbohydrate antigen 199, and carbohydrate antigen 125 levels, were within the NR. Metagenomic next-generation sequencing (NGS) was used to detect HHV-6B sequences in the peripheral blood and ascites, for which the viral load counts were 75944 and 7707 copies/mL, respectively (NR, <1000 copies/mL). Decreased numbers of CD3^+^ T cells (188/μL; NR, 470–3260/μL), CD4^+^ T cells (108/μL; NR, 200–1820/μL), and CD8^+^ T cells (73/μL; NR, 130–1350/μL) were measured via flow cytometry. IL-6 levels were elevated to 85.84 pg/mL (NR, 0–10.3 pg/mL), and those of IFN-γ rose to 15.14 pg/mL (NR, 0–7.42 pg/mL).

**Figure 1:**
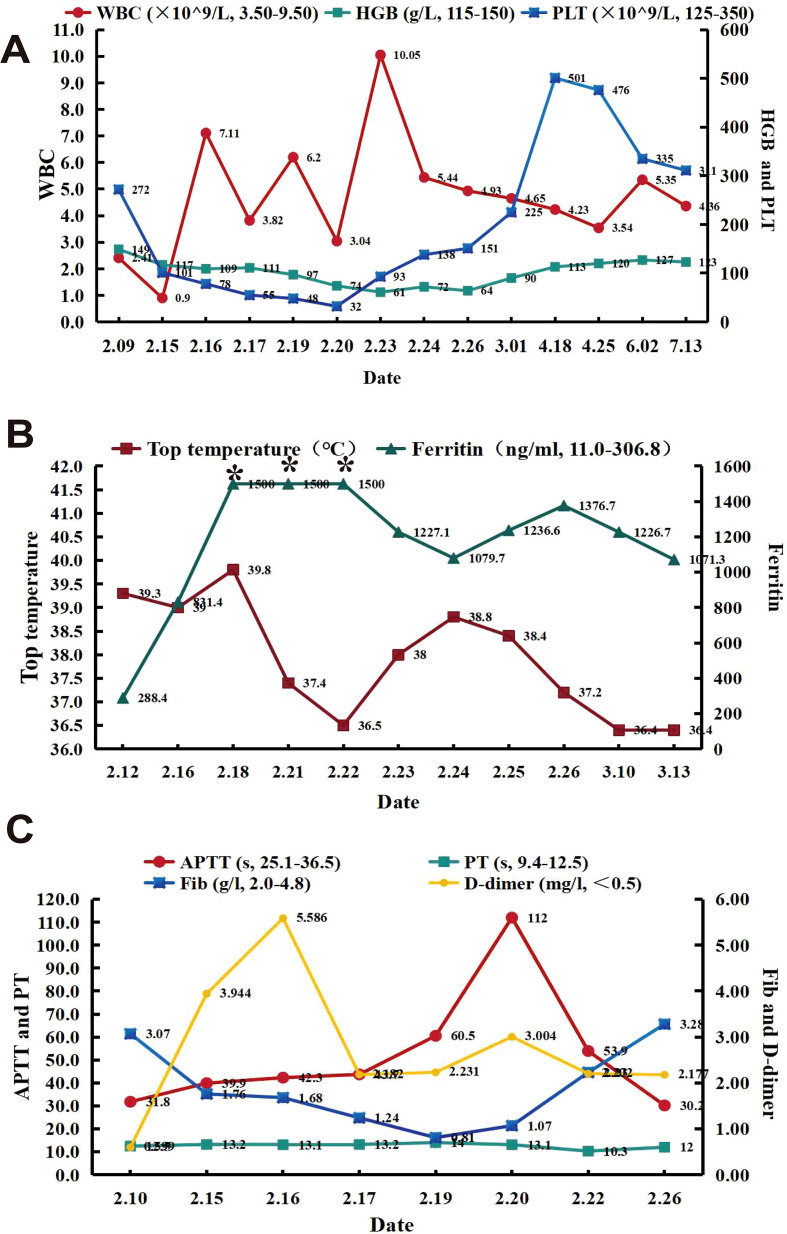
Synopsis of the clinical course. **(A–C)** Dynamic changes in **(A)** blood cell count, **(B)** body temperature and ferritin levels, and **(C)** coagulation function. APTT, partial thromboplastin time; Fib, fibrinogen; Hb, hemoglobin; PLT, platelet; PT, prothrombin time; WBC, white blood cell. *The serum ferritin levels exceed the upper limit of the reference range (>1500ng/mL).

**Figure 2:**
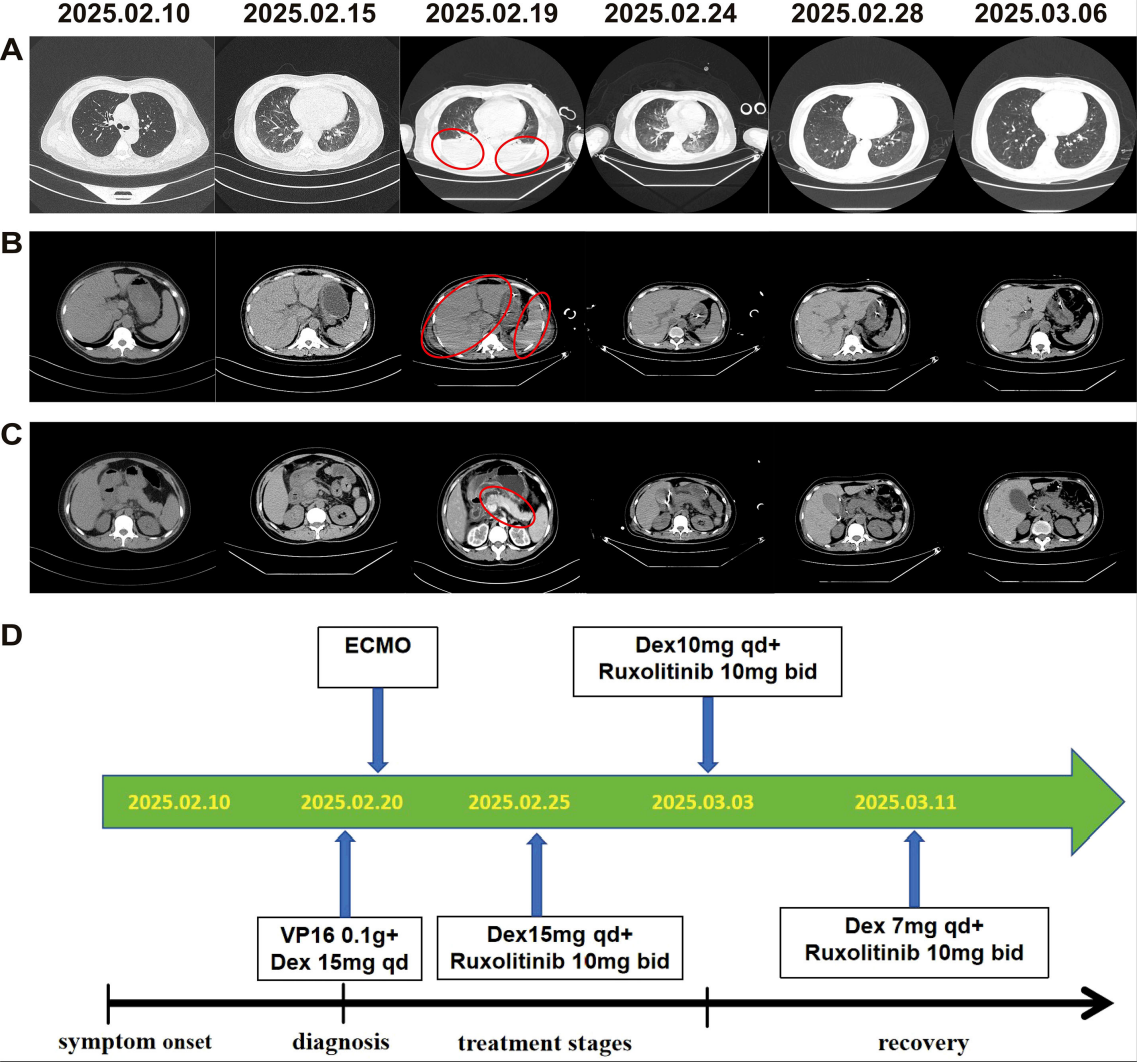
Time course of treatment and computed tomography findings. **(A)** The lung (new infectious lesions and massive pleural effusion, red circle); **(B)** the liver and spleen (enlargement, red circle); **(C)** the pancreas (acute pancreatitis, red circle); **(D)** the timeline of the patient’s treatment. Dex, dexamethasone; ECMO, extracorporeal membrane oxygenation.

**Figure 3:**
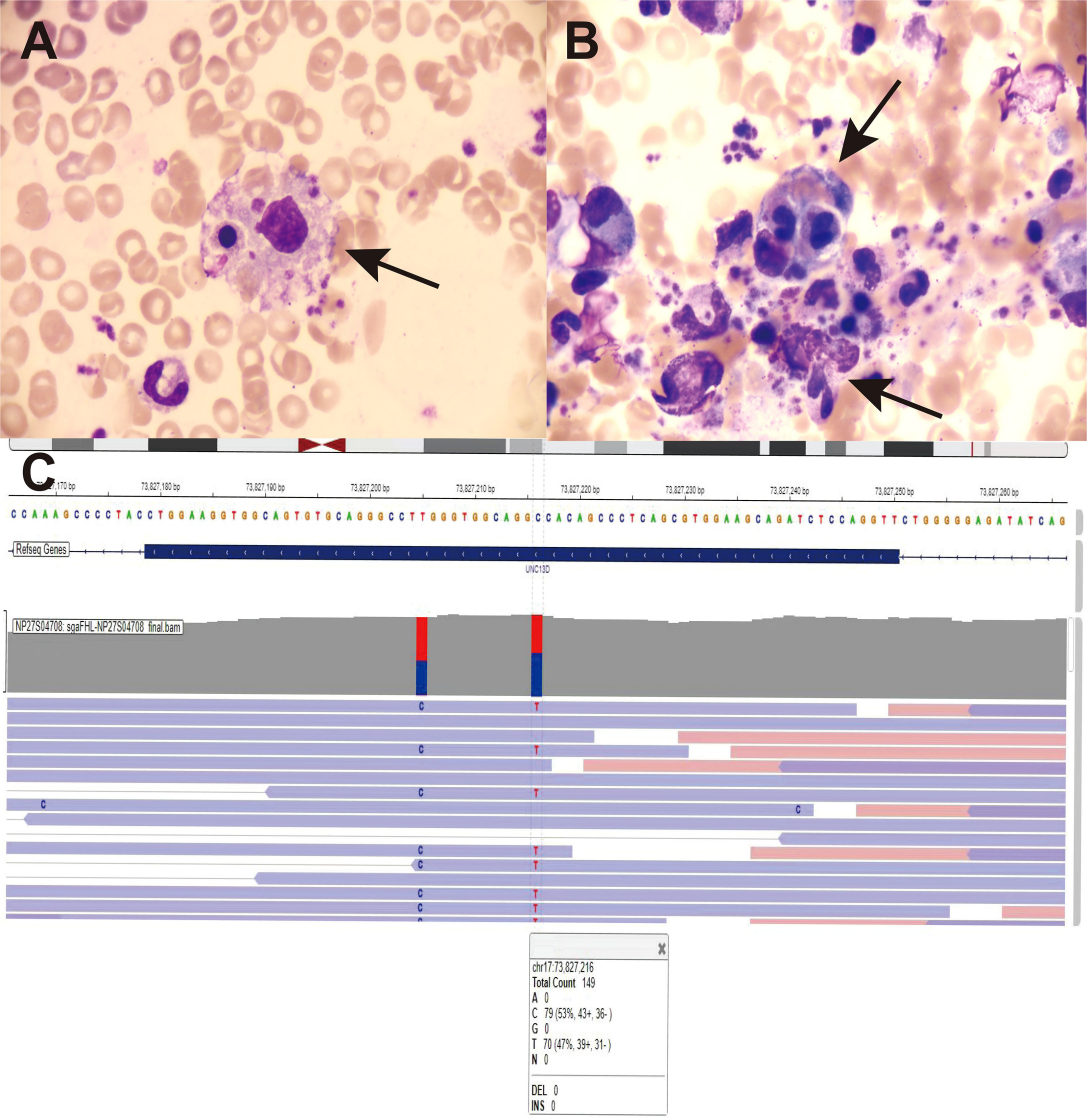
Bone marrow morphology and gene mutation. **(A)** The hemophagocytes are round and have phagocytosed late erythroblasts and platelets (black arrow). **(B)** The hemophagocytes have phagocytosed late erythroblasts, neutrophilic granulocytes, and platelets (black arrow). **(C)** Screen capture from the integrative genomics viewer showing the genetic structure of the identified heterozygous variant, *UNC13D* c.2588G>A/p.G863A, on Chr17.

After admission, the patient received symptomatic treatment, including anti-infection, liver protection, antipyretic, and other supportive treatments, which still could not prevent subsequent disease progression. On admission and during hospitalization, the changes of her white blood cell, hemoglobin, platelet counts, ferritin level, body temperature and fibrinogen level were shown in **Figures 1A–C**. Her computed tomography scans of the chest and whole abdomen were performed at different time points (**Figures 2A–C**). Owing to the unexplained persistent high fever, progressive decline in complete blood count, elevated serum ferritin levels, and progressive hepatosplenomegaly, HLH was suspected. Bone marrow puncture revealed a large number of hemophagocytes that had phagocytosed red blood cells, granulocytes, lymphocytes, and platelets (**Figures 3A, B**). The patient met six of the eight HLH 2004 diagnostic criteria (4). Consequently, the diagnosis of HLH was established on the 10th day after admission. After a multidisciplinary evaluation and communication with the patient, HLH-94 treatments (dexamethasone 15 mg/day, etoposide [VP16] 0.1 g twice a week) combined with gamma-globulin (0.4 g/kg, days 1–5) were administered. The patient was unable to tolerate the side effects of VP16, which led to acute pulmonary edema and respiratory failure requiring treatment with veno-venous extracorporeal membrane oxygenation. Subsequently, VP16 was replaced with ruxolitinib 10 mg twice per day. The therapeutic scheme was shown in **Figure 2D**. The pleural effusion were obviously resolved (**Figure 2A**). The sizes of the enlarged liver, spleen and pancreas were significantly reduced (**Figures 2B, C**). Whole-exome sequencing revealed a heterozygous *UNC13D* c.2588G>A/p.G863A mutation on Chr17 (**Figure 3C**). This *UNC13D* mutation could not be identified in three immediate family members because of the loss of contact over many years.

Her white blood cell, hemoglobin, and platelet counts gradually increased (**Figure 1A**), and her ferritin levels decreased and body temperature returned to normal (**Figure 1B**) after treatment for half a month. Fibrinogen level also gradually normalized during therapy(**Figure 1C**). The patient recovered rapidly and was discharged from the hospital after 40 days, after which she continued to receive an oral ruxolitinib tapering regimen. Follow-up for about half a year showed good recovery, and the patient was able to return to normal activity. The peripheral blood NGS test for HHV-6B virus was negative.

## 3 Discussion

Herein, we described an adult patient who presented with an unexplained fever and multiple organ failure. The patient was admitted to the intensive care unit for treatment due to symptom exacerbation and was ultimately diagnosed with HLH. HHV-6B infection detected via next-generation sequencing in her peripheral blood and ascites, together with analysis of the patient’s medical history, suggested that viral infection was the cause of secondary HLH in the patient; however, a heterozygous gene mutation characteristic of HLH was found upon genetic testing of the patient. Infection-related HLH is the most common form of acquired HLH in both children and adults, with EBV infection predominantly occurring in Asian populations (5). Therefore, testing and screening for herpes virus infection in adult patients during the diagnostic process for HLH is of great significance to assist in the search for the etiology or predisposing factors for HLH. Our patient tested negative for EBV and other viruses but was positive for high HHV-6B loads, as detected via next-generation sequencing.

HHV-6B infects most individuals within the first three years of life and often manifests as roseola (6). Reactivation of HHV-6B is rare and typically asymptomatic in immunocompetent individuals (7). In contrast, HHV-6B is a major cause of opportunistic infections, which lead to life-threatening complications in immunocompromised individuals. However, this is common and associated with a wide range of end-organ diseases in immunocompromised patients (8). Unlike other human herpes viruses, HHV-6B can integrate into chromosomes as a mechanism of latency, which may result in the inheritance of chromosomally integrated (CI) HHV-6B (9). Our patient’s peripheral blood test for the HHV6B virus was negative after recovery. Based on this result, we can exclude the possibility that the individual carries CI HHV-6B. People with CI HHV-6B always have high levels of plasma viremia, regardless of virus reactivation due to cell breakage. Therefore, the patient’s current condition is likely attributable to an acquired HHV-6B infection, rather than the reactivation of chromosomal integration of the HHV-6B virus.HHV-6B hampers the host immune responses by downregulating class I major histocompatibility complex molecules (MHC-I), which impedes the presentation of antigens to CD8^+^ T cells. The downregulation of MHC-I disengages inhibitory receptors on NK cells, leading to their activation and the subsequent killing of target cells when NK cell-activating receptors interact with stress ligands that have been upregulated on those cells. NK cells are known to respond to HHV-6B infection (10), and HHV-6B also participates in the mechanism controlling their activity. Weaver et al. (11) reported that the HHV-6B U20 glycoprotein binds to UL16-binding protein 1, masking it from recognition by NK group 2 member D and interfering with NK cell activation. Rizzo et al. (12) considered that HHV-6B infections have a remarkable effect on the expression of miRNAs and transcription factors, subsequently inducing the impairment of NK cell function and facilitating virus escape strategies. HHV-6B also sequesters the NF-κB p65 subunit to inhibit innate immune responses, including those of NK cells (13). In summary, infection with the HHV-6B virus results in reduced NK cell activity *in vivo*, aligning with the pathophysiological changes observed in HLH.

*UNC13D* is documented to have more than 200 distinct mutations and is a contributing factor to FHL3, in which compound heterozygous mutations occur at approximately 47.6%, homozygous mutations at 36.6%, and heterozygous mutations at 15.6% (14). *UNC13D* homozygous mutations associated with HLH often occur during childhood in primary HLH, which differ from *UNC13D* heterozygous mutations that often occur in adults with HLH, consistent with hypomorphic mutations (15). As shown in **Table 1**, after conducting a literature review spanning nearly seven years, we identified 18 adult patients diagnosed with hemophagocytic syndrome who exhibited mutations in the *UNC13D* gene. All patients were aged 18 years or older, with the youngest being 18 and the oldest 48. Specifically, these patients had single heterozygous mutations without complex heterozygous variants. Regrettably, the literature provided limited additional information, omitting details on viral infections—including those mentioned in the present study, such as EBV and HHV-6B—and treatment protocols. *UNC13D* plays a critical role in the biogenesis, tethering, and priming of cytotoxic vesicles and in the endoplasmic reticulum (16). The monoallelic *UNC13D* c.2588G>A variant could partially impair NK cell cytotoxicity; deficiencies in *UNC13D* genes compromise cytolytic granule exocytosis at the immunological synapse, which impairs the cytotoxic activity of T/NK cells (17).

**Table 1:** Cases of hemophagocytic lymphohistiocytosis carrying the *UNC13D* heterozygous mutation in adults published in last seven years.

Author, year	Sex	Age (years)	Gene mutation in *UNC13D* (type)	Zygosity	Diagnosis
Gu et al., 2025 (19)	Female Male	29 31	c.2588G>A c.2588G>A	Heterozygous Heterozygous	HLH HLH
Bloch et al., 2024 (20)	NA	n = 6, ≥18	NA	Heterozygous	HLH
Xinh et al., 2021 (21)	Female Female Female Male Male Male Female Male	19 36 42 23 21 19 29 31	c.3151G>A c.3151G>A c.965_967>68bp c.2243C>T c.859-3C>T c.859-3C>T c.2296C>T c.118-307G>A	Heterozygous Heterozygous Heterozygous Heterozygous Heterozygous Heterozygous Heterozygous Heterozygous	HLH HLH HLH HLH HLH HLH HLH HLH
Chen et al., 2018 (22)	Male Male	25 48	c.2553 + 5C>G c.115_116insG	Heterozygous Heterozygous	HLH HLH

NA: not available.

HHV-6B infection may influence heterozygous *UNC13D* mutations in individual with HLH through a variety of mechanisms. For example, viral infection may alter the intracellular environment, triggering the production of cytokines and signaling molecules that interfere with the normal expression and regulation of *UNC13D*, thereby accelerating the emergence of disease-causing mutations. Our patient presented with decreased levels of CD4^+^ and CD8^+^ T cell subsets following admission; simultaneously, the levels of inflammatory markers (IL-6 and IFN-γ) increased. During HH6VB infection or reactivation, the virus may trigger the activation of the mutated *UNC13D* gene in patients, leading to decreased NK and T cell activity, release of inflammatory factors, and activation of phagocytes to induce phagocytosis. We hypothesize that if the onset of mutation occurs in *UNC13D*, this will further disrupt the balance of the immune system, increase the likelihood of related diseases, or aggravate the existing condition due to further disorders of the immune system after viral infection.

Allogeneic hematopoietic stem cell treatment should be considered for patients with homozygous *UNC13D* mutations. The adult patient with an *UNC13D* heterozygous mutation reported in the present study is currently healthy and alive after treatment with ruxolitinib and dexamethasone (Ru-D). Zhou et al. reported that the Ru-D regimen had a higher response rate and led to favorable survival outcomes in adult patients with HLH (18). The prognosis of patients with HLH and heterozygous *UNC13D* mutations should be correlated with the age of onset; many subsequent developments can lead to lymphoma. Allogeneic hematopoietic stem cell transplantation is also recommended if a donor is available.

## 4 Conclusion

In conclusion, HHV-6B infection may act as a trigger in patients with heterozygous *UNC13D* mutations, and it may also accelerate the pathogenicity of these mutations in adults with HLH. We suspect that the heterozygous *UNC13D* mutation variant could be conditionally pathogenic in HLH and might be triggered by viruses, such as HHV-6B, or by age-related immune function decline in adults. A limitation of this study is that the *UNC13D* mutation could not be identified in the patient’s three immediate family members due to loss of contact. Another limitation is that it is just a single case. Future studies need to be undertaken involving a larger number of cases, along with virology and genomics correlation.

## Data Availability

The original contributions presented in the study are included in the article/supplementary material. Further inquiries can be directed to the corresponding author.
